# The geopolitics of the European super league: A historiographical approach and a media analysis of the failed project in 2021

**DOI:** 10.3389/fspor.2023.1148624

**Published:** 2023-03-07

**Authors:** Xavier Ginesta, Carles Viñas

**Affiliations:** ^1^ Department of Communication, Faculty of Business and Communication, University of Vic-Central University of Catalonia, Vic, Spain; ^2^ Department of History and Archeology, Faculty of Geography and History, University of Barcelona, Barcelona, Spain

**Keywords:** European super league, geopolitics, sports industry, competitive balance, football (soccer)

## Abstract

**Introduction:**

The main objective of this article is to analyse the reasons why the 2021 European Super League project failed. The authors ask whether, in addition to the popular clamour against a semi-closed competition, it was the combination of geopolitical interests of the different actors currently involved in European elite football that prevented the project from going ahead.

**Methods:**

The main methodological framework is based on a case study, which follows an Stakian approach. To do so, on the one hand, a historiographical analysis of the case has been done; on the other hand, authors have complemented this case study with an analysis of 23 pieces of news published on the website of five mainstream newspapers (from April to June 2021) from those countries with the most significant European football leagues: The United Kingdom (The Guardian), Spain (El País), France (Le Monde), Italy (La Repubblica) and Germany (Der Spiegel). To supplement the analysis of this phenomenon, authors have also considered other relevant news published in other mainstream press or news agencies (such as The New York Times, Politico, The Yorkshire Post, The Times, Marca, Bloomberg and Reuters).

**Results and Discussion:**

The authors conclude that, while financially the Super League debate has not been closed, in defending the current business and competition model of European football, UEFA has had the complicity of owners and shareholders of the founding clubs outside of their traditional historical roots, as well as governments that have made football an asset because of their geopolitical positioning, such as Qatar and the UK post-Brexit.

## 1. Introduction

On 19 April 2021, hours before the European Clubs Association (ECA) approved the new format of the UEFA Champions League (UCL) from 2024, the last attempt by the richest clubs of Europe to give the green light to a European Super League was announced, which would have broken the current competitive pyramid whose main standard is the Champions League. This proposal was led by the president of Real Madrid, Florentino Pérez, and agreed by twelve founding clubs from the Premier League, LaLiga and Serie A: AC Milan, Arsenal FC, Atlético de Madrid, Chelsea, FC Barcelona, Inter Milan, Juventus, Liverpool, Manchester United, Manchester City, Real Madrid and Tottenham Hotspur.

A number of authors have analysed the historical appeals of the most important clubs of Europe to the UEFA to have a greater decision-making power in the distribution of the income generated by the competitions that the UEFA controls (1–4). Moreover, the COVID-19 pandemic, which began in early 2020, negatively affected the economy of clubs, which lost some four billion euros in revenue, compared to pre-pandemic income (5, *p*. 192). At the same time, several authors have also analysed the requests by the clubs to be able to participate in competitions that have greater uncertainty and thus more market value (1, 6–8). Menchén (5, p. 166) remembers the owner of Juventus, Andrea Agnelli, in the context of the World Football Summit (WFS) in 2018, characterising the debate in which the big clubs, FIFA and UEFA are immersed: “The clubs are the only ones taking risks; it used to be true when people said it was only a game, but now it's a business and this needs to be taken into account […] When I look at the cluster of football *stakeholders*, I see leagues, federations and players that don’t take risks, while we are the ones who invest in stadiums, training centres, the development of young players”.

However, two days after announcing the Super League project, the English clubs, Atlético de Madrid and the Milan clubs abandoned the project in the face of criticism from football fans, governments, UEFA and FIFA. The main objective of this article is to analyse and understand the reasons why the European Super League project, promoted by twelve teams of the Premier League (EPL), LaLiga and Serie A in April 2021, failed. We ask whether apart from the popular outcry against a semi-closed competition, it was the combination of geopolitical interests of the different actors (FIFA, confederations, federations, leagues, states and clubs) that currently converge in European elite football that prevented the project from moving forward.

## 2. Literature review

There is a long Anglo-Saxon tradition of studying sport as a social phenomenon, but it was not until the beginning of the 21st century that a broader scientific study of sport emerged. Other disciplines, from various perspectives such as physical activity and sport sciences have been tasked with modestly addressing those aspects that went beyond competition in some academic fields. The global dimension of football has favoured research in areas that were rarely analysed until recently. The History of Sport provides an example (9, 10). Today, the initial Anglo-Saxon tradition has been complemented with other research that has given a more global and interdisciplinary scope (11–16).

A significant research approach focusses on the geopolitics of football (17–23), which examines complex realities based on the comparative analysis of different variables. Recent studies from Chadwick (24, 25) research in depth the synergies among sport, geography, the economy and politics, and advocates “the necessity for the sport community to change its utilitarian-neoclassical framing of sport. Moving forward from here, one contends that sport must be viewed and researched in terms of a geopolitical economy” (25, p. 7). He conceptualizes the geopolitical economy of sport as “the way in which nations, states and other entities engage in, with, or through sport for geographic and politico-economic reasons in order to build and exert power, and secure strategic advantages through the control of resources within and via networks of which sport is a constituent part” (25, p. 9).

Therefore, history, politics, geography and economics appear as keys to understanding specific events or actions, such as the case studied here: the creation of a European Super League in football (26). The emergence of a group of new owners, from outside the world of football, who invested huge sums in clubs for different reasons, stimulated the proliferation of studies that sought to analyse this phenomenon from a geopolitical perspective. This is how, under the umbrella of the advent of millionaires, sheikhs and oligarchs to a game undergoing a process of spectacularisation (2). This line of research was developed in parallel, examining the non-sporting contexts from an interdisciplinary approach akin to international relations (27, 28).

## 3. Theoretical-contextual approaches to the European super league

### 3.1. The background: a historical journey

As an industry that accounts for 21 billion euros annually, football has evolved enormously, whilst remaining rooted in a capitalist rationale (21). This is shown by the constant change that competition structure has undergone to make the game ever more economically advantageous for the most powerful contemporary football clubs. The history of the Champions League itself is a good example: when in 1955 the French newspaper *L'Equipe* conceived the notion of the former European Cup, this project was born without the approval of UEFA, which was only one year old at the time (8).

This being the case, we might question whether a slow but continuous process of Americanisation of the competition-spectacle model exists in Europe, in which the maximisation of profit is the primary goal of the participants (29). The various reforms that the format of the European Cup, known as the Champions League since 1993, has undergone, follow this logic (8). As Holt (4, p. 35) states, the clubs were “increasingly dissatisfied by the level of income accrued by UEFA through the Champions League, and also the manner in which it was distributed”. In the United States, based on a system of closed competitions, drafts are organised, salary limits are imposed and the commercialisation of audio-visual and advertising rights is centralised, taking advantage of the strength of the cartel with the aim of favouring a competitive balance (30).

This is the wider context within which the debate surrounding the creation (or not) of a European Super League of football has been contextualised. Indeed, since 1988, a number of proposals have been put forward, driven primarily by large media groups: Mediaset in 1988, Media Partners in 1998 and News Corporation in 2012 (3, 4, 31). Shortly after the conglomerate founded by Rupert Murdoch articulated its own proposal, Badia reflected that the “the pressure exerted by the large media groups for the big European clubs to leave their domestic league and concentrate on a European Super League has become more present than ever and is gaining ever greater purpose, more based on the notion that the model of such a Super League would provide the participants and owners with significant negotiating capacity to increase their ordinary income (broadcasting*,* marketing and matchday)” (1, p. 22).

Investment funds and banks have also been behind the European Super League project. Holt (4, p. 35) explains how JP Morgan was involved in the Media Partners' attempt to create a Super League in 1998 with an investment of 1.2 million pounds. In this vein, in 2020 reports published by the *Financial Times* that CVC Capital Partners could have been interested in financing a global club super league project were joined, according to Sky News, by an initial proposal with JP Morgan as the main financial partner. The first visible targets were two English clubs: Liverpool and Manchester United. In recent years, two other clubs, Juventus and Real Madrid, have also been particularly active in promoting this new competition. JP Morgan had negotiated financing the promotion group with between 5 and 6 billion euros in order to start the new competition (32).

The launch of the Super League has thus been on the table of the big European football clubs for years, which have been a permanent pressure group to get UEFA to change the structure of the Champions League, to create greater competitive balance and from which they could receive more income for their participation (8). In the 2019–2020 season, the winner of the Champions League won around 111 million euros — out of the 2.04 billion euros that UEFA had to distribute among the participants. In relation to the proposal for the new competition, which was to be funded by JP Morgan, the Spanish newspaper *Marca* suggested that the winner could end up earning some 1 billion euros (33), a figure that would be impossible for UEFA to match, even with the arrival of new sponsors such as Barclays.

On the night of April 19, 2021, hours before the UEFA executive committee approved the new pyramid of its club competitions from season 2024–25, the break was completed. Twelve clubs, led by the President of Real Madrid, Florentino Pérez, took the step and announced this new Super League: AC Milan, Arsenal FC, Atlético de Madrid, Chelsea, FC Barcelona, Inter Milan, Juventus, Liverpool, Manchester United, Manchester City, Real Madrid and Tottenham Hotspur. These big clubs opted for a championship model where there was more competitiveness between the participating teams and an even more commercial approach. This involved implementing a semi-closed competition, which relativised the maximisation of the result, in which there would be fifteen permanent participants and five more that varied depending on their performance in the previous seasons, similar to the way in which the basketball Euro-League already functions, which began in the season 2000–01 and is controlled by the private company Euro-league Basketball, with headquarters in Barcelona. This competition would have to coexist with national leagues (2, 8).

However, two days later, the big six English clubs publicly distanced themselves from the project, citing the public and political pressure unleashed when it became public, at the same time that the prestigious economic news agency Bloomberg reported on a possible new €6 billion funding package for UEFA from the British investment firm Centricus Asset Management (34). One week later, Real Madrid, FC Barcelona and Juventus were left alone to defend, to different degrees, the project. While this may fail, the underlying debate will continue since the structural problems of European football (the economic and competitive imbalance) remain unresolved.

Although the Super League project is recent, it has already attracted academic attention. Just a few months after the failure of the project, different authors began publishing editorials or short commentaries in prominent journals highlighting the research perspectives that it has opened (26, 35–39). Furthermore, in tandem with the Super League project, a PhD dissertation (8) was published highlighting the need to revise the competitive structure of European competitions in order to improve the competitive balance between the clubs.

### 3.2. Competitive balance theory as justification for the super league?

The most common justification among academics for the football Super League is based on the analysis that some researchers, especially from the field of sports economics, have made of the economic and competitive imbalance that exists between the clubs of the major European football leagues (6; 7, 40, 41).

Jordi Badia also makes an interesting contribution from the communication sciences with the following summary (1, p. 4): “The position of these authors has oscillated between those that believe that the current situation of combining the UCL with the domestic leagues is optimal, and those that consider that the richest clubs should compete exclusively in a European Super League of American format”. Badia aligns himself with the latter, because he understands that those who defend the combination of the European and domestic competitions fail to take into account an essential factor: “The decisive weight that the competitive balance of the championships has on the clubs’ economic viability” (1, p. 4).

On the one hand, this author concludes that as soon as these clubs take part in the UCL, the revaluation of [their] intangibles becomes as, or more, important than the immediate revenue that the continental competition generates (1, 8). On the other hand, this fact adds to the growing sporting difference between the main European clubs and the other participants in domestic competitions. According to Badia (8), who conducted a longitudinal analysis of results between 1996 and 97 to 2020–21 seasons, after the *Bosman* ruling and the rise of satellite televisions (Pay-TV) between 1994 and 1995, the economic inequality among the clubs is a progressive and unstoppable trend. Hence, economic inequality increases the difference in sport performance among clubs. “This difference in sport performance affects the competitiveness of the domestic leagues. It is only a matter of time, therefore, that this loss of competitiveness ends up affecting the interest of the spectators and, consequently, the commercial value of both, domestic leagues and their clubs, decreases” (8, p. 163).

Based on competitive balance theory, FIFA is the first to endorse the fusion of competitions in emerging markets: the possible African Super League (42), the merger of the leagues of Mexico and the US or of Belgium and the Netherlands (2). Furthermore, “continental Super Leagues, if organised by club consortiums and not by confederations, would provide the perfect feeder tournaments for its own flagship competition, which it could then brand, promote and sell as “The Best”, short-circuiting the existing continental cups (slow-killing them, in fact), and thereby weakening the economic and political power of confederations, two of which [Gianni] Infantino's FIFA perceives as a threat to its desire of total dominance, South American CONMEBOL and European UEFA” (42). With regards to the European football competitions, things change when a new championship that escapes the control of UEFA is proposed globally in Europe, which is considered the cradle of modern football (21).

## 4. Methodology

Given the different considerations involved, and since the phenomenon is a very recent one that has as yet to generate a substantive bibliography, the only viable focus of study is a multidisciplinary perspective. In fact, without the geopolitical perspective (19, 20, 25, 27, 28, 43, 44), it would be difficult to understand, beyond the strictly sporting and economic sphere, the magnitude of the European Super League project. Thanks to this approach, we will be able to explain the causes, the effects and the implications of the announcement of the competition and the reasons for its initial failure.

Consequently, this article follows an intrinsic case study approach (45). Stake (46, p. 136), calls a study an intrinsic case study “if it is undertaken because, first and last, the researcher wants better understanding of this particular case”. To do so, “Stake suggests the use of observation, interviews and document review in qualitative case study research” (47, p. 143). Although the Stakian perspective recognizes the use of analysis protocols “that help [researchers] draw systematically from previous knowledge and cut down on misperception,” he gives precedence to intuition and impression rather than guidance of the protocol (45, p. 72).

On the other hand, to report the “researchers” dressing of the case's own story” (46, p. 144), in addition to an historiographical approach, we analysed 23 pieces of news published on the websites of five mainstream newspapers from those countries with the most prominent European football leagues: the United Kingdom (*The Guardian*), Spain (*El País*), France (*Le Monde*), Italy (*La Repubblica*) and Germany (*Der Spiegel*). The news items selected were published between April and June 2021, as it was during this period that the most relevant reactions in favour and against the project took place: it comprises the period between the presentation of the Super League and the resignation of 9 (out of 12) of the founding breakaway clubs. Out of all the articles published by these newspapers during that period, we selected those news items that were written following this themes' matrix: geopolitics, economy, football and Europe.

Finally, to complement the case study story, we have also considered other relevant news stories published in other mainstream press or news agencies (such as *The New York Times*, *The Yorkshire Post*, *The Times*, *Marca, Politico,* Bloomberg and Reuters).

## 5. Results and discussion

This case study (the downfall of the 2021 Super League project) is presented in four sections, according to the following key conclusions identified in the news analysis carried out: the influence of fans as guarantors of traditional football values, the clash between the Super League promotors and Qatar's geopolitical interests, the role of current football governing bodies (FIFA and UEFA) and, finally, post Brexit British public diplomacy strategy. Following an intrinsic case study logic (45), theory, literature, and data are combined to explain the trends underpinning the narrative.

### 5.1. The fans: guarantors of the values of football?

The announcement of the creation of the European Super League provoked all sorts of reactions. As well as the institutional reactions (federations, governments and clubs), fans showed their dissatisfaction of what they saw as a further step in the commodification process of football (48, 49). However, opposition from fans was due to the colonisation of the commercial forces of football and their response to the authoritarian movements that govern football, visible in slogans like “Against modern football” (50) — a phenomenon that was significantly accentuated during the first half of the 1990s, leading to the gradual uprooting of fans with respect to their teams. The 1996 UEFA European Football Championship in England, for example, attracted several companies that saw the tournament as an opportunity to make themselves known through advertising campaigns amounting to £100 million, to which should be added the £45 million received from television rights and the £305 million in four years that Sky had agreed to broadcast the English Premier League (EPL) from 1992 (51, 52).

The arrival of foreign owners, together with the growing interest of both large companies and the middle class, which led to higher ticket prices as both demand and the cost of the product-spectacle (the football match) increased. This also impacted upon this process of alienation that eroded the fans' sense of belonging, some of whom were excluded from the stadium for not being able to afford the new policy of ticket and season ticket prices. Their marginalisation, however, was also influenced by the remodelling of the stadiums of these new “consumer cathedrals” (50, p. 81), and the disappearance of the terraces imposed by the *Taylor Report* in 1990 as a consequence of the rise in hooliganism and the Hillsborough tragedy that occurred the previous year with nearly one hundred fatalities.

All this transformed the appearance of the stands and was symptomatic of the parallel break-up in the community structures of the working class, consolidated by the establishment of the welfare state. Not only did the fans suffer the consequences of the establishment of the football industry, many clubs were also affected, with some even forced to dissolve. Thus, while between 1982 and 2010 there were 67 cases of insolvency, during the 2010–11 season alone there were 36 (51, p. 37).

In the United Kingdom, where from the end of the nineteenth century football became the favourite pastime of the working class, and the clubs' links with their respective communities were tightly-kit, this distancing and the process of turning fans into consumers led to a number of important consequences. These included the departure of Manchester United fans following the purchase of the club by the US tycoon Malcolm Glazer to create a new team, FC United of Manchester (49, p. 216), the appearance of the first supporters' trusts, such as the Northampton Town Supporters' Trust (NTST) created to raise funds to prevent the club from disappearing, and the AFC Bournemouth Trust Fund, together with the emergence of Supporters Direct in 2000, set up with the aim of helping clubs have owners rooted in the community (50, 53). In fact, this pressure group stimulated the creation of over a hundred supporters' trusts, such as Wimbledon FC, Exeter City, Chesterfield and Brentford (51). These developments also interested the fans of EPL clubs, such as West Ham United, Liverpool and Arsenal, who tried to coordinate an approach to deal with the emergence of large investors through the Independent Supporters Association (ISA). Whist the influence of these platforms has been limited due to the smaller number of shareholders, most of them have positioned themselves as guarantors of the traditional soul of their respective clubs.

This was precisely what happened with the announcement of the Super League project, which many fans understood as one more step in the erosion of their clubs' identity. The morning after the news broke, thousands of fans gathered at the gates of their respective stadiums to express strong feelings of rejection. The protests were limited to banners and chanting against the clubs' owners, except for the Manchester United fans who forcibly entered Old Trafford to invade the pitch in a more visible display of their anger. According to Greater Manchester Police, 1,200 fans participated in this demonstration (54). At the same time, a number of fans blocked the entrance to the Lowry Hotel where Manchester United players were staying, thus preventing them from arriving at Old Trafford in time to play their match against Liverpool, which was eventually postponed (55). Some fans even unfurled banners calling for the establishment in English football of the Bundesliga 50 + 1 rule, which ensures that the ownership of the clubs (with a few exceptions), lies in the hands of the supporters as majority shareholders. This practice has itself been recently called into question by the Bundeskartellamt (BKartA) (56), the independent competition regulator.

A few hours later, the English clubs withdrew from their agreements and announced that they were abandoning the Super League project. For several days, the protests persisted in Manchester with the aim of pressuring, unsuccessfully, members of the owner family to surrender control of the club. The protests in Britain contrasted with the “lukewarmness” –according to Santiago Segurola (57) — of the reaction of the Spanish fans. We can point to three causes for this lukewarmness: (a) the deep political connections between Real Madrid's President, Florentino Pérez, and the Spanish political authorities (57); (b) the historical lack of coordination among the Spanish supporter groups due to deep ideological rivalries; and (c) the negative economic effects of the COVID-19 pandemic on the budgets of Real Madrid and FC Barcelona (58). The economic breakdown of European football caused by the pandemic (59) was also a factor that divided opinion within the Italian *tifosi* regarding the Super League project (60, 61). On the one hand, the Super League is considered to be an inevitable consequence of the commodification process of football, whereas on the other hand it is conceptualised as an elite project led by entrepreneurs that signals the “death of calcio”, becoming a juggernaut against “*il calcio è del popolo*” [grassroots football] (62).

In conjunction with the ambivalent and even hostile reaction of fans, there was the withdrawal of sponsors (as announced by the Swiss watch brand Tribus in respect of Liverpool and MyProtein with regard to Manchester United), the fall of the clubs' share price on the Stock Exchange, and the confirmation of the aforementioned funding of UEFA by the British Centricus Asset Management (63).

### 5.2. The super league clashes with qatari interests

The 2021 Super League project was a lame duck from its inception without the presence of two of the current leading clubs in European football: PSG and Bayern Munich. Both clubs have shared connections with the Emirates of Qatar, an actor that has been instrumental in the development of the football industry in recent years, hosting the 2022 FIFA World Championship (64–73).

Acting as host to the World Cup has been one of the most important milestones in the process of place rebranding that the Emirates began back in 1995 (72, 74). It involves a geopolitical positioning strategy in which sport has been one of the most valued assets and serves as a seminal example of what is known as “sport place branding” (75), or what could be articulated as the search for a competitive global/regional identity through sport. Brannagan and Reiche (72, p. 55) explain how “in the realm of global sport, Qatar's niche soft power strategy rests on three key pillars”: the hosting of sports events; leveraging domestic excellence and, finally, overseas sports investments, as suggested by the following examples.

Within this dichotomy, one year after Qatar was awarded the 2022 FIFA World Cup, the sovereign fund Qatar Sports Investments (QSI) bought PSG (2011) and, consequently, the Paris-based club became the main European ambassador for the Emirates. As Chanavat and Desbordes (70, p. 221) conclude, the acquisition of PSG by Qatar “is part of a “real” cohesive strategy (marketing, geo-marketing, geopolitical). It frames a process of investment in all directions, industrial, sports and media of Qatar”. It should be noted, however, that Nasser Al-Khelaïfi, the President of PSG and beIN Sports television channel, has an ongoing case in Switzerland for corruption, and needed to avoid conflict with FIFA knowing both the importance of the 2022 World Cup for the repositioning of the Emirates brand, and the fact that Qatar Airways has been one of the main partners of world football's governing body since 2017. For example, the Qatari airline company was one of the main sponsors of Euro 2020 (76).

Moreover, the sport place branding strategy deployed by Qatar has resulted in significant overseas investment in sports sponsorship (77, 78). In addition to the partnership between the Qatari Tourist Development Agency (QTA) and PSG (70), the most important example was that which linked Qatar Sports Investments (QSI) to FC Barcelona, which enabled the Qatar Foundation and Qatar Airways to be the main sponsors of the team's shirt between 2010 and 2017 (2). However, Qatar Airways has closed out other sponsorships, including with PSG and Bayern Munich. While Bayern's general manager Karl-Heinz Rummenigge warned that, more even more than fending off the Super League elite football clubs had to streamline their budgets, it is indeed the case that Qatar Airways has advertised in the Allianz Arena, Bayern's stadium, since 2016, and the club has a contract with the company, until 2023, so that the logo also appears on the team's shirt. Furthermore, Bayern has expressed its support for modifying the 50 + 1 regulations in Germany, in order to give more leeway to the entry of private investors.

The proximity of the 2022 World Cup was likely to prove an inconvenience when consolidating the Super League project, due to the fact that the founding clubs had to publicise it quickly before the official endorsement, by the ECA, of the proposal for the new Champions League format from 2024 to 25 onwards, which expands it from 32 to 36 teams. Regarding the UEFA European competitions, *La Repubblica*, *Le Monde* and *Der Spiegel* highlight the interests that beIN Sports has in these tournaments: The channel has invested 500 million euros in broadcasting rights for the Middle East, North Africa, Hong Kong, Malaysia, Brunei and Singapore (76, 79, 80).

When the World Cup or the European Championships take place, politics carries almost more weight than economics. In respect of Qatar, which has sought to exploit the mediatisation of the tournament as a key part of its Sport and Cultural Diplomacy strategy, despite the negative scrutiny the country experienced after having been confirmed as host (72, 81, 82). As merely one example of this; on February 23td, 2021 *The Guardian* exclaimed: “Revealed: 6,500 migrant workers have died in Qatar since World Cup awarded” (83). Ginesta (2) reminds us: “Hosting the football World Cup was surrounded by controversy from the first moment that Qatar won the rights against the United States”. Criticisms from the start, such as the extreme weather conditions in the summer –which meant it had to be moved to November and December–, the political instability of the Middle East and the denunciations by various NGOs of civil rights violations in the Emirates, were accentuated in the summer of 2019 by alleged irregularities in its awarding after the arrest of former UEFA president Michel Platini, who was accused of peddling influence between the Emirates and the French government, when Nicholas Sarkozy was President. Sepp Blatter, Ex-President of FIFA, Michel Platini and Mohamed Bin Hamman, the head of Qatari football, were involved in this episode, which the French newspaper *L'Equipe* called “Qatargate” (84, pp. 107–108).

Conversely, for European states, which have traditionally controlled the clubs under the umbrella of national federations and their participation in the current competitions, realise that they could also control the symbolic narrative that sport generates (85). International team championships are a great showcase for national flags and stereotypes and, in fact, in the shadow of this symbolic universe linked to the representation of the nation-state that is exhibited at every World Cup or European Nations Cup, it was also impossible for the players of the twelve founding clubs to be prevented from playing in these competitions, despite the threats of UEFA and FIFA after the Super League project was announced.

### 5.3. UEFA and the leagues: seeking global business stability

With the COVID-19 pandemic under relative control, Euro 2020 was played a year later (in the summer of 2021). Amongst the main sponsors of the event was the Qatari Emirates, using the Qatar Airways brand. In addition, taking advantage of the executive congress of the European Club Association (ECA) during the European Championship, the UEFA President Aleksander Ceferin and the Qatari businessman Nasser Al-Khelaïfi, elected head of the ECA after the resignation of Andrea Agnelli following the failure to consolidate the Super League project, reaffirmed their rejection of the Super League, agreeing that UEFA and ECA would work together to forge a new *Memorandum of Understanding* (MoU) to ensure the long-term stability of European football (after 2024) (86). The aim is to seek financial improvements so that clubs end their lack of liquidity and to agree with FIFA a better schedule of international matches, thus ensuring that players receive the necessary protections when participating in competitions with their respective national teams.

The movements of the governing bodies of international football, as well as the managers of the main nation league championships, sought to ensure their control of the football industry and, therefore, to prevent clubs from increasing their decision-making power and, above all, their income. To achieve this, they did not hesitate to coerce and threaten, with varying degrees of subtilty, that the clubs involved in the Super League project would face potential sanctions on players or possible exclusion from tournaments. UEFA, for example, decided to withhold 5% of the revenue from the six English clubs, Inter Milan, AC Milan and Atlético de Madrid, which is the amount they should receive for the competitions they took part in for a year –a light punishment given the agreement they reached when they signed the “Club Commitment Declaration”. In contrast, the process of sanctions against the “rebel” clubs was halted due to the pre-emptive measures included in an interlocutory order issued by the Madrid Court No. 17 that explicitly prohibited the UEFA and FIFA from retaliating against Real Madrid, FC Barcelona and Juventus, the three defiant clubs that still supported the project, as well as their managers and footballers (87, 88).

Within the national competitions, one of the harshest critics of the project was Javier Tebas, President of La Liga. To the head of the organising body of the Spanish championship, the notion that FC Barcelona, Real Madrid and Atlético de Madrid should stop considering the domestic championship as one of their main priorities would constitute a genuine economic catastrophe. The current value of LaLiga television rights is over 1.8 billion euros per year. However, the launch of the Super League, if it really sought to be valued in respect of broadcasting revenue, would have to consider competing with the national tournaments for the weekend television slot (2, p. 117).

The consequences for the national leagues of going ahead with the Super League explains the belligerent response of organisers in seeing their business endangered. One of the most energetic critics was, as mentioned, Javier Tebas, who in a series of statements to the media called the promoters of the Super League instigators of a “Coup against European football” referring to the three clubs that stayed faithful to the project (Real Madrid, FC Barcelona and Juventus). He later added that it all seemed like “an idea cooked up in a bar at five in the morning”. Speaking at the Foro Sport Business Days, held in June 2021, Tebas even insinuated that if Real Madrid and FC Barcelona persisted with their stance, the percentage they both receive for television rights could be reduced.

For its part, the English Premier League was also in favour of financially sanctioning the clubs initially involved in the Super League with a £22 million fine. In the event of any recidivism, the EPL threatened the six English teams with an individual fine of £25 million and the loss of 30 points in the league standings (89). This reflects the establishment of coercive measures to deter any new project in the future that attempts to break the current business controlled by the Premier League.

In the case of Italy, it was the other clubs that demanded sanctions for Juventus, Inter Milan and AC Milan. Through a letter addressed to the president of the Lega, Paolo Dal Pino, 11 Serie A teams, the same clubs, with the exception of Parma and Cagliari, which had opposed the transfer of the television rights of the tournament to Dazn, urged the calling of an extraordinary assembly to address the imposition of sanctions on the three clubs citing “the evident and serious damage” that their involvement in the project meant for transalpine football (90).

All this confirms that the current structure of the football industry is not only based on competitive criteria, but also brings together a significant set of geopolitical and economic relationships between the actors involved (clubs, competitions, federations, states and sponsors) that determine the configuration and organisation of major sporting events (2, 24, 75). Sport and politics, while they might be considered separate in an ideal world, constantly converge in real life (82). However, as Murray points out (2019: 135), in defining the global dynamics of sports diplomacy, there is a tendency for the large organisations that manage the governance of international sport to seek stability within the system (91).

### 5.4. Football and the “great glory” of Britain

The dynamics of British politics might also have been one of the factors that hindered the latest Super League project. We might consider whether the opposition to the European Super League was exploited by the then Prime Minister Boris Johnson in two areas: in the field of domestic politics to strengthen voter loyalty; and in international politics in order to retain the Premier League as one of the most important assets for its sports diplomacy (92–95).

In this vein, Stephen Castle (96) told *The New York Times*: “For Prime Minister Boris Johnson, the crisis has presented a rare opportunity to seize the moral high ground on an issue that matters to many of the voters who helped him to a landslide victory in the 2019 election”. Fear that the Super League crisis could affect the loyalty of the Conservative vote in regions that were supportive in 2019 has been interpreted as one of the reasons why Downing Street opposed the proposal, especially bearing in mind that the districts of Greater Manchester, Merseyside, South Yorkshire and West Yorkshire were among the most contested between Labour and Conservatives in the 2019 General Election. The Prime Minister was able to appear before the public as a defender of the working class, who are connected to the historical origin of many of the country's clubs, as well as an opponent of the arrival of foreign capital, despite his government's reluctance to legislate in favor of a system of club ownership similar to that of the Bundesliga, where the aforementioned 50 + 1 rule prevails. At the same time, it would not be the first time that some of these clubs have set the agenda for domestic politics. As Castle (96) recalls: “Invoking his own poor childhood, [Manchester United footballer], Marcus Rashford galvanized a campaign against child poverty, and ultimately forced Mr. Johnson to change policy over free school meals. This week the boot was on the other foot as Mr. Johnson was able to condemn the super league plans before Mr. Rashford, whose club initially signed up to the proposals”.

In contrast, at a time when Boris Johnson's government was strengthening the sense of belonging to a “free” nation with the wish to build a “more global United Kingdom” — as Johnson said when the Brexit trade agreement was ratified by the European Parliament on 28 April 2021 (97) — the Premier League still remained one of the most important promotional assets of his country overseas. In fact, in March 2021 the UK government published the document *Global Britain in a Competitive Age. Integrated Review of Security, Defence, Development and Foreign Policy* (98), a text that seeks to position the strategy of the British government in the new post-Brexit and post-Covid 19 geopolitical context. According to McClory (99), “the Integrated Review explicitly underlines the importance of soft power to the UK and recognizes the role that assets like the British Council, the BBC World Service, top-ranking universities, museums, tourism, heritage and sport play in delivering greater prosperity and international influence for the UK”.

The Premier League is not only the football competition with the most value for the television market (2, p. 117), but is also a symbol that combines tradition and the reinforcement of the “sense of place” of its participants, with the necessary commodification of the sport inherent in the process of globalisation. Boris Johnson highlighted that football was “one of the great glories of this country's cultural heritage” (100), and he used a Covid-19 press conference to condemn the idea that clubs could be “dislocated from their home cities, taken and turned into international brands and commodities that just circulate the planet, propelled by the billions of banks, without any reference to the fans and those who have loved them all their lives” (100).

The British government took a further step in June 2021. On 10 June, *The Times* published an exclusive stating that, “overseas players and managers at Premier League clubs who take part in breakaway competitions such as a European Super League will have their work permits revoked under new rules” (101). In this regard, the Home Office agreed to the FA “changing its rules in light of the attempt to form a breakaway Super League in April so that the organization can withdraw its Governing Body Endorsement (GBE) needed for work permits” (101). However, we might surmise that when the UK regains full political sovereignty, it cannot lose control of any of its national symbols. In fact, coincidentally, the Super League project was falling apart when Bloomberg published that “UEFA is in discussion with Centricus Asset Management over a six-billion-euro financing package to overhaul its flagship soccer tournament” (34).

Besides the UK positioning against the Super League, inside the European Union France and the European Commission were the first political voices to criticise the project. “In France, where Qatari-backed behemoth Paris Saint-Germain have reportedly refused overtures to join the money-spinning breakaway competition, [Emmanuel] Macron said the proposal ‘threatened the principle of sporting merit’”, reported *Politico.* European Commission Vice President, Margaritis Schinas refused the project by arguing that the current model of competitions is a “values-driven model” and the Super League would be a project of “rich and powerful clubs” (102). Spanish and Italian Governments echoed the EU and French positioning a few hours later (103), following the wish of the French Government to be at the forefront of the European leadership and political agenda after Brexit. However, while the Italian Government refused the project using arguments in favour of meritocracy in sport, the Spanish Government was worried about how the Super League could clash with the local competition (LaLiga) and this could affect the performance of the Spanish national team (104). In Germany, with its own model of club ownership, the government did not interfere. It was the German Football Federation (DFB) which openly refused the project defending a competition model based on sporting merits.

## 6. Conclusions

The Super League project of April 2021 shook the football scene from top to bottom. Although the project was called into question two days later, the possibility that twelve of the most important European clubs could break the competitive pyramid and embrace a project on their own helped to trigger a debate that had long been necessary (4).

Beyond highlighting that these twelve clubs seek to find a formula to increase their income when the pandemic has affected their economies (59), a review of the current structure of competitions is inevitable if we look at two essential variables: the economic inequality between clubs; and the subsequent competitive imbalance that has been generated since the 1990s (8). Furthermore, while UEFA and the national leagues have doubled down in order to stop the twelve founders leaving, FIFA is supporting similar projects on other continents. We should therefore note that while FIFA President Infantino did not wish to trigger a confrontation between FIFA and UEFA, he had his own agenda and strategy in order to maximize FIFA's control of world football (105).

Whilst justification for the Super League is inevitable in the medium term, or for what could be a major revision of the revenue redistribution criteria of the current model of the Champions League; thereby requiring an analysis that follows an interdisciplinary approach. The failure of the latest attempt to create a European Super League cannot be merely reduced to the impact of the protest by a group of fans rising up against the tycoons that promoted it. According to the case study, the reasons for the failure of the Super League project must also be searched for in the current dynamics that mark the geopolitics of football (25): the interests of FIFA 2022 World Cup host, Qatar; the power relations network between football governing bodies and the French Government, the public diplomacy strategy of post-Brexit Britain and the political interests of the Italian and Spanish Governments. In fact, it is the convergence and tension between local and global interests among the actors involved in the Super League project that has hindered its implementation.

Furthermore, the Super League debate has exposed the hypocrisy within contemporary football. Professor Paul Widdop, from Manchester University, gave an in-depth analysis of this during an interview in *The Yorkshire Post*. At a time when North American capital has taken control of some English teams, it is logical for football to explore models of spectacularisation similar to that of the NBA or NFL. Investment requires stability, leading to the near certainty that “a Super League would be huge, it would rival the NBA, the NFL, there's no doubt about it. We are so outraged now, and yet you, me, every fan that has watched Sky Sports and paid money for replica shirts is complicit in this move towards where we are going” (106).

In brief, what has been made clear in the ephemeral project of the European Super League promoted by the English Big Six, the Spanish trio of FC Barcelona, Real Madrid and Atlético de Madrid, and the Italian clubs Juventus, Inter Milan and AC Milan, under the auspices of the investment bank JP Morgan, is that when defending the current business model and football competition in Europe, UEFA had the collusion of owners and shareholders from the founding clubs outside their traditional historical roots. Paradoxically, the origin of these football guarantors for the old continent is extra-European: Asians, North Americans, Russians and, above all, from the Middle East where Qatar is a seminal actor in understanding the current configuration of the sports industry (73, 77, 84).

This research suggests future lines of inquiry in critically explaining this phenomenon. Moving beyond studies relating to the competitive balance of competitions (6; Szymanski and Kuypers, 1999; 1, 7, 8, 40, 41), of particular interest is the advance in the comparison between a revision of the European competition model and examples of future supranational competitions in Africa and North America. This will further enable us to explore any similarities or differences with the Chinese Super League (107).

## Data Availability

The original contributions presented in the study are included in the article, further inquiries can be directed to the corresponding author.
